# Accurate and semi-automated reassociation of intermixed human skeletal remains recovered from bioarchaeological and forensic contexts

**DOI:** 10.1038/s41598-021-99962-x

**Published:** 2021-10-12

**Authors:** Ioanna Anastopoulou, Fotios Alexandros Karakostis, Katerina Harvati, Konstantinos Moraitis

**Affiliations:** 1 Department of Forensic Medicine and Toxicology, School of Medicine, National and Kapodistrian University of Athens, 75 Mikras Asias Street, 11527 Athens, Greece; 2 DFG Centre of Advanced Studies “Words, Bones, Genes, Tools”, Eberhard Karls University of Tübingen, Rümelinstrasse 23, 72070 Tübingen, Germany; 3 Paleoanthropology, Senckenberg Centre for Human Evolution and Palaeoenvironment, Institute for Archaeological Sciences, Eberhard Karls University of Tübingen, Rümelinstrasse 23, 72070 Tübingen, Germany

**Keywords:** Biological anthropology, Bone

## Abstract

Commingled remains describes the situation of intermixed skeletal elements, an extremely common occurrence in contemporary forensic cases, archaeological mass graves, as well as fossil hominin assemblages. Given that reliable identification is typically impossible for commingled contexts, a plethora of previous studies has focused on the development of refined methods for reassociating the bones of each individual skeleton. Here, a novel virtual approach for quantifying the degree of three-dimensional shape compatibility between two adjoining bone articular surfaces is put forth. Additionally, the integrability of this method with traditional osteometric techniques is evaluated. We focus on the paradigm of the hip joint, whose articulating bone elements (the femur and the innominate bone) are crucial for reconstructing the biological profile of unidentified human remains. The results demonstrate that this new semi-automated methodology is highly accurate both for large commingled assemblages (such as those resulting from mass disasters or burials) as well as smaller-scale contexts (such as those resulting from secondary burials).

## Introduction

A primary aim of anthropological sciences is to reconstruct the identity, characteristics, and/or living conditions of humans that lived in the past. To approach these, researchers study the three-dimensional (3D) form of human bones, relying on an integrative methodological toolkit that may encompass the use of traditional morphometrics, bone histology, 3D virtual reconstructions and analyses, as well as advanced biostatistics^1–3^. However, a reliable application of these methods is often impossible due to variable taphonomic factors that affect the preservation status of human remains and/or their anatomical position within the context of recovery. One of the most critical consequences of taphonomy is “commingling”, a term describing an assemblage of mixed bone remains corresponding to different individuals.

Since the accuracy of osteological methods depends on the combined use of several anatomical regions, commingled contexts constitute a critical limitation for osteological studies. Such intermixing of human bone remains may form due to a variety of external factors. In forensic contexts, this is typically the consequence of mass disasters, such as natural phenomena (e.g., earthquakes, fires, tsunamis), war, mass murder (e.g., terrorist attacks), or accidents (e.g., plane crushes, car accidents)^4–6^. In bioarchaeology, commingling is often associated with mass graves such as those associated with past pandemics like the Black Death, battlefield remains, shipwrecks, or mass executions^7,8^. Finally, in paleoanthropological research, commingling is also a typical caveat that prevents the thorough and accurate analysis of some of the most crucial fossil-bearing contexts worldwide^9–11^.

The treatment of commingling requires the reassociation of each individual’s bones, after the important step of distinguishing human from animal skeletal remains^12^. Given that the application of DNA analysis in commingled contexts has serious limitations, such as postmortem DNA degradation, high financial cost and destruction of the bone specimens, the reassociation is usually performed by using anthropological methods. Traditionally, the most common method involves visual sorting. According to this basic approach, articulating bones are segregated based on their resemblances in surface coloration, texture, general robusticity, or matching pathological lesions^13^. However, this practice has been extensively criticized for its questionable accuracy as well as for largely relying on the observer’s level of experience^14,15^. Moreover, this process can be greatly time-consuming and impractical to apply in larger scale contexts (i.e., multiple individual skeletons), where there can be multiple potential combinations for each pair of articulating bones in context. Over the last few decades, the advent of osteometric techniques for reassociating adjoining bones^16,17^ and the development of protocols integrating metric and visual sorting practices have led to a considerable increase of accuracy in contexts with a small number of intermixed individual skeletons^14,18,19^. Nevertheless, the error of such approaches continues to be substantial, especially for large-scale contexts^14^.

A series of recent studies have introduced new 3D virtual anthropological techniques that allow for the reliable pairing of an individual’s left and right bilateral bones (i.e., “pair-matching techniques”), an important step in sorting commingled skeletal remains. Karell et al.^20^ applied the mesh-to-mesh value comparison (MVC) on 3D bone models. They evaluated the similarity between two skeletal elements by digitally comparing their entire three-dimensional geometry. Although these researchers state that the automated part of their methodology needs improvement, their results were promising. Fancourt et al.^21^ utilized a 3D elliptical Fourier analysis function and Hausdorff distances between the specimens in order to sort skeletal elements. This 3D shape comparison is only applicable in intact skeletal elements and bilateral bones (bones of the same type but of different anatomical side). Garrido-Varas et al.^22^ applied a technique combining generalized Procrustes analysis and multivariate statistics for pairing left and right metacarpals. Their excellent results underline the value of incorporating geometric morphometric techniques in sorting commingled human remains. However, despite the promising findings of the above 3D approaches for pair-matching bilateral elements, no virtual anthropological methods have been developed for sorting each anatomical side’s different bones by individual skeleton (e.g., the femur with the innominate bone). In fact, even in a recent pilot study by De Simone and Hackman^23^ relying on 3D models, no virtual anthropological method was employed. Instead, these authors applied the standard οsteometric protocol of Byrd and Adams^16^ directly on the 3D scans. Their findings showed a compromise of the model efficacy, further highlighting the fact that new methods of sorting designed for 3D models are needed. Given that the process of sorting different (articulating) bones by individual comprises a fundamental step for any analysis of mixed skeletal contexts^12^, the absence of such virtual anthropological methods represents a major and crucial gap in osteological research and practice.

In this framework, the present study puts forth a novel, precise, and semi-automated methodology for reassociating commingled human remains, focusing on the level of 3D shape compatibility between adjoining bone articular surfaces. This approach shows high levels of accuracy for reassociating one of the most informative anatomical region of the human skeleton, the bone elements of the hip joint (i.e., the innominate and the femur). The anthropological analysis of these particular bones is crucial for reconstructing an unidentified individual’s biological profile, as it can provide reliable assessments of biological sex, age-at-death, stature, and approximate body mass^12,24^. Furthermore, due to the hip’s increased vulnerability to certain types of accidents (e.g., falling, especially for older individuals), this anatomical region may often preserve important paleopathological information (lesions) regarding the identity of an individual and his or her living conditions.

## Results

As described below in Materials and Methods, our approach assesses the probability that a femur and an innominate bone may belong to the same individual by assessing the degree of shape compatibility between their two adjoining articular surfaces (i.e., the femoral head and the acetabulum). This method relies on the selective digitization and analysis of 3D landmarks on points of the two opposing surfaces that come in contact when they virtually interlock (articulated hip). We have confirmed the repeatability of our landmark placements by computing the percentage error per landmark (for more details, see Materials and Methods). Across all 11 landmarks and semilandmarks, the average intra-observer error was found to range from 0.34 to 1.11%, while the inter-observer error varied between 0.53 and 2.12%.

**Figure 1:**
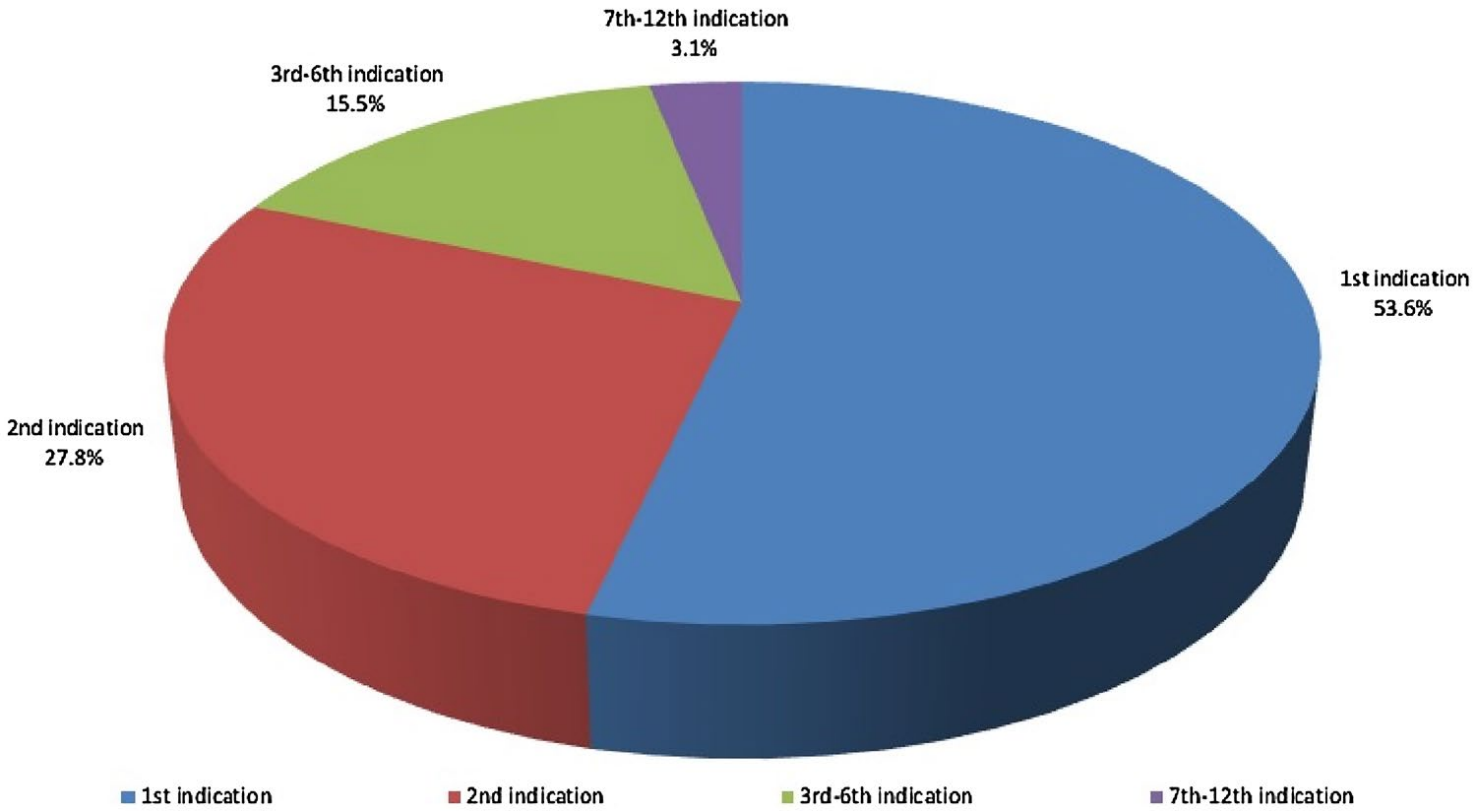
Correct sorting rates for a large-scale sample of 97 individuals. The methodology’s “1st indication” is considered to be the femur with the smallest shape difference (i.e., Manhattan distance; see Materials and Methods) to the examined acetabulum.

This new landmark-based approach was applied on a highly diverse reference sample of 97 individuals from different geo-chronological contexts, sexes, as well as age-groups. The average accuracy error in identifying the adjoining bones that belonged to the same individual was 1 ± 2 individuals (i.e., *circa* 1% ± 2% of the sample). Overall, in 81.4% of the sample, for each single innominate bone (acetabulum), this method would typically identify the matching femur within its first two indications (i.e., the femora with the two smallest Manhattan distances, see Materials and Methods). More specifically, as Fig. 1 illustrates, more than half of the cases (53.6%) presented no error, as the correct match of the examined acetabulum was the method’s first indication. In 27.8% of the cases, the correct match was the method’s second indication (out of all 97 individuals), while in 15.5% of the cases the correct match was included among the first 3 to 6 indications maximum. Finally, the number of cases for which the correct match was listed among the first 7 to 12 indications was less than 3.1%. The aforementioned rates were calculated for the whole sample of 97 individuals, however, when calculated by population the differences across the three did not exceed 3%.

The above accuracy rates were further evaluated for small-scale commingled contexts through a series of three blind tests, each involving a total of ten artificially commingled individuals (see Materials and Methods). The application of the present virtual approach as a stand-alone technique led to a 10/10 correct sorting in blind test #1, while 9/10 commingled femora and acetabula were correctly reassociated in blind tests #2 and #3 (Table 1). Additionally, for these three tests, it was attempted to combine the method presented in this study with an osteometric sorting technique previously developed by our research group^18^. According to this process (see Materials and Methods), for each acetabulum, the two femora with the smallest shape difference (i.e., smallest Manhattan distances) were further examined metrically using a function of Anastopoulou et al.^18^. The combination of the techniques produced almost perfect results (Table 1). In two of the blind tests (#1 and #3), all artificially commingled acetabula and femora were correctly reassociated (10/10). In blind test #2, one of the innominate bones was not possible to be attributed to the person it corresponded to, as the metric method falsely rejected both of the possible matches (one of which was correct, indicated by our 3D shape approach). In sum, across the artificially commingled samples of the three tests, the mean error of the landmark-based approach was 0.67 individuals (SD: 0.58), while the combination of the method presented in this study with an osteometric sorting technique presented a mean error of 0.33 individuals (SD: 0.58).

**Table 1:** Blind tests results.

Method	Blind test #1	Blind test #2	Blind test #3
GM only	10/10	9/10	9/10
GM and metrics	10/10	9/10	10/10

“GM only” signifies that the first indication of the present methodology was considered to be the match, while “GM and metrics” signifies that the first and second indication of the present methodology were supplementary examined metrically.

## Discussion

The sorting method introduced here shows high accuracy rates for reassociating both large (i.e., 97 individuals of our entire sample) and small-scale (i.e., three subsets of 10 individuals each) commingled contexts. For almost 97% of the cases (94 out 97 individuals), the matching bone was identified within the first six indications, which translates to a successful exclusion rate that ranges from 94% (i.e., 91 of the 97 false matches excluded) to 100% (no error). Importantly, this exclusion rate was even greater for the smaller-scale contexts of our three blind tests (Table 1). On this basis, we argue that the proposed landmark-based method comprises a powerful tool for sorting human skeletal remains from archaeological and forensic contexts. Essentially, an accurate excluding power of 94–100% is able to pinpoint a few remaining candidate specimens (up to 6%). This can be further filtered out via standard visual or osteometric sorting techniques (see Anastopoulou et al.^14^), a fact that highlights the importance of combining different methodologies in order to achieve better sorting results. For instance, the three blind tests of this study demonstrated that combining the landmark-based approach with osteometric sorting can maximize accuracy (Table 1).

The sample’s great diversity in terms of geo-chronological context and characteristics suggests that the present methodology is not considerably affected by inter-population variation, sex, age or body size effects. The differences in accuracy rates among populations examined were minimal. Particularly, the differences across the three populations in these rates (Fig. 1) did not exceed 3%. Therefore, we argue that this approach is applicable to human remains of unknown origin or identity. In a paleoanthropological context, future research could investigate the accuracy of this approach on other species, potentially supporting its applicability for commingled fossil hominin assemblages^25,26^. In such intermixed contexts, reliable sorting of bones by individual could help expanding the sample sizes available for study, potentially providing new insights into human evolution.

Furthermore, such an extensive reduction of each bone’s potential matches in large-scale contexts also renders the use of DNA matching feasible, assuming that genetic material is adequately preserved. In contrast, the application of such genetic analyses is often highly impractical and costly for commingled contexts that may involve thousands of bone elements corresponding to an often unknown number of individual skeletons.

The reassociation of innominate bones and femora is of crucial importance, as their combined presence in an individual’s remains can provide the basis for reconstructing its basic biological profile. In particular, innominate bones are traditionally utilized for sex determination^27^ and age estimation^28^, while femora can provide accurate estimation of stature^29^, body mass^30^ and sex^31^. On this basis, future applications of this approach on commingled contexts including these bones could readily provide a minimum number of individuals with assessed biological profiles (sex, age, and body size parameters), offering critical insights into the profile and identify of the skeletal assemblage under study, facilitating subsequent genetic analysis.

**Figure 2:**
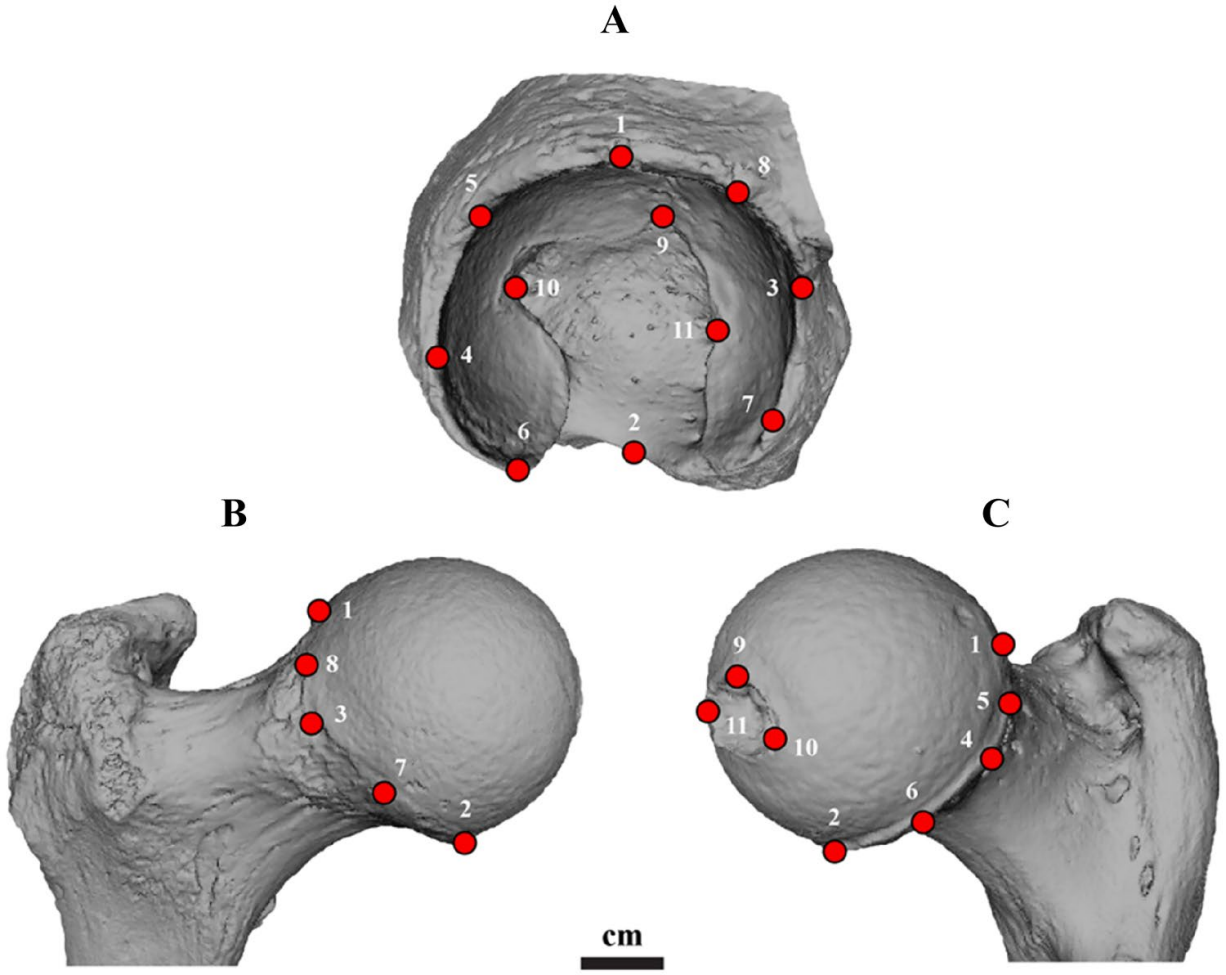
Landmark placement. The landmarks of the acetabulum (**A**) are approximate mirrored counterparts of the landmarks of the femur (**B**-anterior view, **C**-posterior view) when the two articular surfaces adjoin. The landmark and semi-landmark descriptions are presented in Table 2.

A major advantage of methods relying on articular surfaces (rather than dimensions of the entire bone) is that they do not require extensive bone integrity. In all anthropological contexts, bone fragmentation is rather common. Many of the existing methods for sorting demand intact or well-preserved bones. The proposed method only requires an adequate preservation of the articular surfaces for its application. In fact, even if a considerable part of the articular surface itself is fragmented, the method will still be applicable if the taphonomic damage does not happen to extend to the 11 distinctive locations of the landmarks digitized (see Fig. 2). This fact further increases the likelihood of the approach’s applicability to fragmented commingled contexts at a level that is unique among existing osteological methods. Moreover, metric methods attempt to reassociate articulating skeletal elements based on their overall size compatibility. Consequently, it is impossible for them to sort individuals of similar size and robusticity, which are quite frequent in commingled contexts^19^. The present study’s methodology is not affected by bone (or articular surface) size, as the raw coordinates of the digitized landmarks were transformed to Procrustes coordinates, a process that removes the effects of size and position^32^.

Future studies should focus on the extension of the present methodology to other adjoining skeletal elements. In particular, the application of this approach to other important joints (e.g., knee, shoulder, elbow) could provide an avenue for semi-automated holistic reassociation of commingled human skeletons. Furthermore, we recommend the application of this methodology to large-scale commingled contexts that were not possible to be reassociated until now based on osteometric or visual sorting techniques, as well as to the extension of our method to skeletal elements of other species in paleoanthropological or zooarchaeological contexts. Importantly, future applications of our method to other contemporary and archaeological population groups are required to further evaluate the low influence of inter-population variation on its efficacy. Finally, it is worth noting that the degree of bone commingling is affected by the degree of primary and secondary deposition of elements into a given mixed context. In cases with only primary deposition, additionally considering the factor of the skeletal elements’ spatial distribution (i.e., proximity in space) could further improve the accuracy rates of our approach. This is because the bone elements that are found to be nearest to each other within a mass grave/deposition are expected to have the highest probability of belonging to the same individual.

## Methods

### Skeletal material

The osteological sample utilized for the present study derives from various and distinctive geo-chronological contexts, so as to ensure that the method is robust against the potential effects of the multiple factors driving inter-population variability (e.g., genes, ecology, living conditions, and lifestyle), and can thus be applied to different recovery contexts, where the identity of the human remains is unknown. The sample consists of 97 individuals of three different populations: contemporary Greeks, medieval Greeks and medieval German populations. The study sample was randomly selected in order to simulate a typical commingled recovery context. It consisted of 52 males and 45 females. They were all complete skeletons (over 90% of all their bones were present), adequately preserved, and of various body sizes. The age-at-death of the known identity individuals ranged from 24 to 92 years, while the bioarchaeological samples included individuals of all three estimated age-groups (young to old adults)^33,34^. The maximum femoral head diameter, measured in mm, was utilized as a body size indicator. In particular, the aforementioned measurement’s range was 15.43, the mean was 44.89 and the standard deviation was 3.83, overall representative of typical human population^35^. More specifically, a number of 33 individuals (17 males, 16 females) were chosen by current forensic cases of the Forensic Anthropology Unit, Department of Forensic Medicine and Toxicology, School of Medicine, National and Kapodistrian University of Athens, Greece. All individuals were of known identity or identified. Additionally, a number of 12 individuals (7 males, 5 females) belonging to the Byzantine cemetery of Abdera (fourth–tenth century BC) were also utilized for the present study^33^. These individuals are housed in the Anthropological Museum of the National and Kapodistrian University of Athens. Finally, 52 individuals (28 males, 24 females) from Grevenmacher (Luxembourg) cemetery site (fourteenth–fifteenth century AD) were included in the study^34^. Individuals of the later population are housed in Human Osteology Collection of the Paleoanthropology working group, Institute of Archaeological Sciences, Eberhard Karls University of Tübingen, Germany. All 97 individuals were formerly buried in individual graves; therefore, it was known which skeletal element belong to which individual.

### 3D scanning and reconstruction

The skeletal elements of the hip joint of the aforementioned 97 individuals were scanned with a handheld 3D scanner (Artec Space Spider™, Artec 3D, Luxembourg). Hip joint is a ball-and-socket articulation, formed by the cup-like acetabulum of the innominate and the almost spherical femoral head. The skeletal elements of the right anatomical side were selected to be scanned. In cases where the right elements were not preserved, the left ones were scanned and reversed via Thermo Scientific™ Amira-Avizo™ Software (version 2019.2). All the skeletal elements’ scans were extracted and saved as “.ply” type of files.

The “.ply” files were imported into the open-access software Meshlab (version 1.3.3, CNR-INC, Rome, Italy) in order to crop and filter the bone models. Only the innominate’s acetabulum and the proximal end of the femur were selected by each 3D model. The remaining anatomical parts of the innominate bones and femora were virtually cropped, so that the 3D models would be easier to handle (i.e., manually rotate). As far as the filtering process is concerned, we utilized the “discrete curvatures” algorithm that colorizes the bone surface according to various discrete curvatures computed. This process is useful for optimal visualization of the articular surface edges that allowed easier identification of areas (as they are colored differently) and thus digitization of landmark points. For the present study, all landmarks are located at the rims of the selected areas (acetabular rim and acetabular fossa, femoral head, and fovea capitis).

### Landmark placement

A total of 11 landmarks were chosen to be placed on both the articular surfaces at the superior, posterior, anterior, and medial projecting points of their rims, as well as at the mid points between them (semi-landmarks). More specifically, for the acetabulum, a number of four fixed landmarks (1–4) and four semi-landmarks (5–8) were manually digitized along the rim, via the IDAV Landmark software package (version 3.6, Institute for Data Analysis and Visualization group, University of California, Davis). In cases that lipping (bony outgrowth at the margin of the articular surface) was present in places where a landmark should be, the landmark was then placed in the internal part of the rim. In addition to the landmark points located at the acetabulum’s lip, another three fixed landmarks (9–11) were placed along the acetabular fossa. The landmark and semi-landmark definitions of the acetabulum are provided in detail in Table 2, while their graphical depiction is presented in Fig. 2.

**Table 2:** Landmark and semi-landmark descriptions of the acetabulum (1–11) and the femur (1–11).

Landmark/semi-landmark	Description
**Acetabulum**	
1	The most superiorly projecting point of the acetabular rim
2	The mid-point of the acetabular notch
3	The most anteriorly projecting point of the acetabular rim
4	The most posteriorly projecting point of the acetabular rim
5*	The mid-point between landmarks 1 and 4
6*	The mid-point between landmarks 2 and 4
7*	The mid-point between landmarks 2 and 3
8*	The mid-point between landmarks 1 and 3
9	The most superiorly projecting point of the acetabular fossa
10	The most posteriorly projecting point of the acetabular fossa
11	The most anteriorly projecting point of the acetabular fossa
**Femur**	
1	The most projecting point of the femoral head rim, when viewed superiorly
2	The most projecting point of the femoral head rim, when viewed medially
3	The most projecting point of the femoral head rim, when viewed anteriorly
4	The most projecting point of the femoral head rim, when viewed posteriorly
5*	The mid-point between landmarks 1 and 4
6*	The mid-point between landmarks 2 and 4
7*	The mid-point between landmarks 2 and 3
8*	The mid-point between landmarks 1 and 3
9	The most superiorly projecting point of the fovea capitis
10	The most posteriorly projecting point of the fovea capitis
11	The most anteriorly projecting point of the fovea capitis

The symbol (*) refers to semi-landmarks.

As far as the head of the femur is concerned, a total of 11 geometrically corresponding landmarks were digitized (Table 2 and Fig. 2). As demonstrated in Fig. 2, the landmarks of the acetabulum constitute approximate mirrored counterparts of the landmarks of the femur. Therefore, when the two bones articulate together in anatomical position, the two sets of landmark points coincide in space. The latter fact was further confirmed by virtually aligning the two bones in articulation.

After the landmark placement was completed for all 97 individuals of our sample, to confirm the procedure was repeated for another two times for five random individuals of the sample by IA and FAK, in order to evaluate both the intra- and the inter-observer reliability of our protocol. The mean deviation and percentage error of the three repetitions were calculated for the raw co-ordinates of each landmark. Afterwards, the average percentage error per individual was computed^36,37^. The aforementioned methodology for error estimation was selected as it distinctly calculates each landmark’s error and thus allowed us to assess the repeatability for each landmark separately. It must be highlighted that the placement of the 11 geometrically defined landmark points does not require any specialized training, as it relies on a standard anatomical orientation of the two bones and the identification of basic morphological features (see Fig. 2). As stated above, we further confirmed this fact through an inter-observer repeatability analysis involving two co-authors from different institutions and areas of expertise (i.e., IA and FAK).

Furthermore, it is worth clarifying that the application of the proposed virtual methodology for sorting mixed skeletal contexts does not require an extensive amount of time for a researcher with basic skills in virtual anthropology, as long as a fast 3D surface scanner (such as the one used in this study) is available. During our analyses and inter-observer test, we calculated that the entire process for each pair of adjoining bone elements (i.e., one individual skeleton) lasted about 11 min (i.e., 1 min of 3D scanning using the Artec Space Spider and without capturing texture information, 2 min of scan post-processing, 1 min for importing the 3D surface models to the landmarking software, 6 min of landmark digitization, and 1 min of copying the coordinates to the statistical software). At such a pace, a commingled context of 30 individual hips could potentially be analyzed within about 6 h of work. This calculation was also confirmed in our three blind tests of 10 individuals each (see above), which required approximately 2 h of work.

### Statistical processing

After the placement, the 3D model landmark raw coordinates were saved in “.pts” format and imported into the PAST software package (version 4.03;^38^). In order to determine the most likely matching femora for each acetabulum (innominate bone), we compared its shape to that of all femora (excluding from the comparison the remaining acetabula). This process was repeated separately for all 97 acetabula of the sample.

In particular, the first step was the Procrustes superimposition of the landmark co-ordinates. The raw co-ordinates of all femora of the sample and the acetabulum under examination were transformed into Procrustes coordinates. Thus, the data were translated, rotated to major axis and scaled. The scaling procedure eliminates the specimens’ size differences. Initially, the analysis was performed without scaling as we considered that the factor of size might add valuable information that can help towards the goal of correct matching. Nevertheless, this approach did not lead to improved results because it is extremely common for individuals with extremely different articular shapes to present almost identical dimensions, which greatly impacts the calculation of inter-landmark Euclidean distances in space. As an example, the landmark configurations between two (almost) identically large individuals will always be much closer to each other in Euclidean space than the configuration of any articular surface that has an identical shape with one of the two, but it is only very slightly smaller than expected. Due to this effect, before scaling in our analysis, we observed that numerous specimens with extremely different articular shapes were initially considered belonging to the same individual (i.e., they presented small Manhattan distances) just because they happened to be of almost identical surface size. Therefore, the factor of size had to be removed for our accuracy rates to highly increase, following the standard geometric morphometric process of Procrustes superimposition^32^, which results in the calculation of Procrustes landmark coordinates.

Following the above scaling procedure, the Manhattan distances between all specimens were calculated in Procrustes shape space. We calculated shape variation between each acetabulum and all femora using Manhattan distance (i.e., the sum of the absolute values of the differences of the coordinates) over the simpler “Euclidean” distance (i.e., the square root of the sum of the squares of the differences of the coordinates), considering the recommendations of previous research^39^. The femora which showed the smallest Manhattan distances (or alternatively the biggest similarity index) relatively to the acetabulum under examination were considered to be possible matches.

### Blind tests

In order to further explore the applicability of the present methodology in small-scale commingled contexts, three skeletal assemblages of artificially commingled (and randomly selected) innominate bones and femora were created. Each assemblage included skeletal elements that belonged to ten random individuals of the initial sample of 97 individuals. The blind test sample #1 exclusively included individuals from the contemporary Greek population, the blind test sample #2 from the medieval German population and the blind test #3 was mixed. The three blind tests were performed in order to test whether the methodology is applicable to samples that are considerably smaller than 97 individuals (i.e., our original sample) and to explore whether the inter-population variation affects the results. For each blind test sample the Manhattan distances of each acetabulum from all the femora of the sample were calculated, as described above.

First, in order to evaluate the landmark-based approach as a stand-alone technique, we spotted the specimen that best matched in shape (between all the ten femora) with the one examined (each acetabulum). The error of the method in each sub-sample and the mean error of the three sub-samples were estimated. Second, to test whether the integration of this new approach with osteometric sorting can further improve accuracies, the two femora which presented the closest distance for each acetabulum examined (i.e., the higher shape resemblance) were additionally evaluated based on metric dimensions. For this purpose, following Anastopoulou et al.^18^, two linear measurements were performed using the “measuring tool” of Meshlab: the Maximum Femoral Head Diameter (FHD) and the Maximum Diameter of the Acetabulum (ODA). As our research group previously described^18^, the simple linear regression equation “ODA = 4.725 + 1.027*FHD ± 2.54” (all units in mm) can metrically reassociate commingled human femora and innominates at a considerable accuracy. The specimens that matched both in shape and size were considered to belong to the same individual and thus it was finally estimated whether combining the present landmark-based methodology with osteometry (by applying metric analysis on the 2 potential candidates) could identify the correct match.

### Ethics declarations

No experiments were conducted for the purposes of this study, which focused on shape analyses of human skeletal remains from various geo-chronological contexts. The present study has been approved by the boards of the research institutions housing each of the anthropological samples used in the analyses (i.e., Medical School of National and Kapodistrian University of Athens and Eberhard Karls University of Tübingen), which are officially and legally responsible for the curation and scientific study of these human skeletal remains. All applied analytical methods were not invasive for the bones and they were in accordance with national state laws and university regulations concerning the scientific study of human skeletal materials.

## Data Availability

The data of this study are available upon reasonable request.
